# Colon-to-hind paw cross-organ sensitization is partially mediated by TrkB.T1-facilitated spinal neuroinflammation to activate lumbar DRG neurons

**DOI:** 10.64898/2026.07.25.740685

**Published:** 2026-07-28

**Authors:** Parshva Mehta, Namrata Tiwari, Cristina Smith, Shanwei Shen, Taggert Barton, Aron H Lichtman, Liya Y Qiao

## Abstract

Patients with bowel disease can develop referred somatic pain at a later time with unknown molecular mechanisms. Using experimental mice with colitis induced by intracolonic installation of 2,4,6-Trinitrobenzenesulfonic acid (TNBS), we find an increase in the percentage of hind paw primary afferent neurons expressing Piezo2 or calcitonin gene-related peptide (CGRP), which are attenuated by TrkB.T1 knockout (KO). Concomitantly, TrkB.T1 KO also attenuates colitis-induced hind paw mechanical hypersensitivity and pain. Next, we find that TrkB.T1 is expressed in spinal cord astrocytes and its expression level is increased by colitis. TrkB.T1 KO reduces colitis-induced upregulation of Tumor necrosis factor-alpha (TNF-α) mRNA but not upregulation of interleukin (IL)-6 mRNA in the spinal cord. Using calcium (Ca ^2+^ ) imaging and ex vivo approaches, we find that TNF-α elicits Ca ^2+^ transients in capsaicin-sensitive as well as capsaicin-insensitive DRG neurons, and increases Piezo2 and CGRP expression in DRG neurons via distinct signaling pathways. Notably, TNFα-or colitis-induced Piezo2 upregulation in L4 DRG neurons is mediated by or associated with the PI3K/Akt pathway that does not participate in CGRP upregulation. In contrast, CGRP upregulation in hind paw primary afferent neurons is associated with an upregulation of phosphorylated cAMP response element binding protein (p-CREB). Finally, we find that TrkB.T1 KO does not change the expression level of transient receptor potential cation channel subfamily V member 1 (TrpV1) in L4 DRG in colitis, explaining the ineffectiveness of TrkB.T1 KO on colitis-induced hind paw thermal hyperalgesia. These results suggest complex and distinct molecular pathways in colitis-induced somatic pain modalities and provide information for specific pain modality management.

## Full Text Availability

The license terms selected by the author(s) for this preprint version do not permit archiving in PMC. The full text is available from the preprint server.

